# Supplementary material for: Atacama Large Aperture Submillimeter Telescope (AtLAST) science: Our Galaxy
Source: Open Res Eur. 2025 Feb 10;4:112. Originally published 2024 Jun 5. [Version 2] doi: 10.12688/openreseurope.17450.2 (PMC11462128; doi:10.12688/openreseurope.17450.2)
Supplement: Peer review history

# Reviewer report: Reviewer response for version 1

**DOI:** 10.21956/openreseurope.18857.r44420

**Published:** 2024-10-08

**Authors:** Tayyaba Zafar

**Affiliations:** 1 Macquarie University, Sydney, New South Wales, Australia

The work outlines the current limitations in the study of our Galaxy emphasising the need for advanced telescope. The authors outline the current shortcomings very well. For advancing galactic studies by bringing together diverse areas like chemical and dynamical surveys, mapping magnetic fields from cloud scales to individual star-forming cores, mapping column density structure of the Galactic plane, dust continuum and planetary disc evolution, are essential. For this, AtLAST will enable to probe the Galaxy's 6D structure.

 Though it is a strong case for AtLAST and clear scientific needs that cannot be addressed by current facilities, the case study can provide more details on technological feasibility, atmospheric stability, cost-benefit and its analysis, and complementary strategies. There is a good comparison with ALMA and ALMA 2030 upgrade, and I may have missed how to avoid redundancy and how these capabilities integrate into larger survey efforts. 

 In future, collaborative strategies with existing data centres could add value.

 In summary, a 50m single-dish sub-mm telescope is a transformative leap to explore Galactic ecosystem.

Is the case presented with sufficient detail to be useful for teaching or other practitioners?

Partly

Is the work clearly and accurately presented and does it cite the current literature?

Yes

If applicable, is the statistical analysis and its interpretation appropriate?

I cannot comment. A qualified statistician is required.

Are all the source data underlying the results available to ensure full reproducibility?

Not applicable

Are the conclusions drawn adequately supported by the results?

Yes

Is the background of the case’s history and progression described in sufficient detail?

Yes

Reviewer Expertise:

Interstellar medium studies, instrumentation

I confirm that I have read this submission and believe that I have an appropriate level of expertise to confirm that it is of an acceptable scientific standard.

# Reviewer report: Reviewer response for version 1

**DOI:** 10.21956/openreseurope.18857.r42607

**Published:** 2024-09-27

**Authors:** Guochao (Jason) Sun

**Affiliations:** 1 Northwestern University, Cook County, Illinois, USA

The authors present a careful and comprehensive case study for the AtLAST 50-m single-dish telescope concept on the scientific themes related to our Galaxy. The paper is overall well-written, providing useful information of broad interest to observational studies of the Galactic Plane as an integral part of AtLAST's expected scientific return. 

 Motivations and background information about AtLAST and each of the identified, Galactic Plane-related scientific themes are provided with sufficient details, with references to the current literature properly included. Advances and novelties to be made by AtLAST are carefully explained and justified with the necessary technical details. As a result, the conclusions on AtLAST's significance and usefulness to advance our overall understanding of the Milky Way are drawn in a way that is well supported by the information provided. 

 The paper in general already meets the criteria of being approved. As detailed below, I only have a few minor comments for the authors to consider in a revised version of the paper. 

 I. It seems that the majority of the discussion in this case study focuses on the Galactic Plane, which of course is where most of the Galactic science opportunities lie. However, the case study is titled "Our Galaxy" and (sub-)mm observations of star formation and the ISM at high Galactic altitude are also of great interest. One good example is the impact of polarized Galactic dust emission on the search of primordial gravitational waves at high Galactic altitudes. Another example is stars located in the Galactic halo (or the thick disc), whose origin remains controversial. It might be useful to briefly mention/comment on these aspects (especially what improvements might be enabled by AtLAST) to give a more complete picture of the scientific return from studying our Galaxy. 

 II. High angular resolution and sensitivity are used to justify the need for a 50m telescope at multiple places in the paper, including a comparison drawn with the 6m FYST. However, it is not clearly stated why other large (sub-)mm telescopes with similar diameters do not meet the same needs. This may be an issue of wavelength coverage, since AtLAST can operate at shorter wavelengths than e.g., 50m LMT and 45m Nobeyama. The authors should more clearly state what factors distinguish AtLAST from these other large, single-dish telescopes. 

 A few other small things: 

 1. Page 6: first paragraph, "a concept for a 50-m... a wide field of view and throughput"; should it be "and high throughput"? 

 2. Page 6: third paragraph, "Compared to recent breakthroughs provided by high-resolution studies..."; some references should be given. 

 3. Page 10: Section 2.2, first paragraph, for what this is worth, CSO has been decommissioned from Mauna Kea but will soon be moved & rebuilt also at the Chajnantor Plateau. The new telescope is named Leighton Chajnantor Telescope (LCT). 

 4. Page 10: Section 2.2, third paragraph, "Toltec on the LMT"; should be TolTEC instead.  

 5. Page 13: Section 3.1.1, last paragraph, "These molecules are able to..."; it's unclear what molecules are referred to here. 

 6. Page 13: Section 3.2, "with |b| < l^{\circ}"; should this be 1^{\circ} instead of l^{\circ}?

Is the case presented with sufficient detail to be useful for teaching or other practitioners?

Partly

Is the work clearly and accurately presented and does it cite the current literature?

Yes

If applicable, is the statistical analysis and its interpretation appropriate?

Not applicable

Are all the source data underlying the results available to ensure full reproducibility?

Not applicable

Are the conclusions drawn adequately supported by the results?

Yes

Is the background of the case’s history and progression described in sufficient detail?

Yes

Reviewer Expertise:

computational astrophysics focusing on high-redshift galaxy formation and evolution; observational cosmology (esp. at NIR and sub-mm/mm wavelengths)

I confirm that I have read this submission and believe that I have an appropriate level of expertise to confirm that it is of an acceptable scientific standard.
